# COVID-19 Pneumonia and Dengue Fever Coinfection in an Individual From Southeast Asia

**DOI:** 10.7759/cureus.18846

**Published:** 2021-10-17

**Authors:** Shaalina Nair

**Affiliations:** 1 Internal Medicine, California Institute of Behavioral Neurosciences & Psychology, Fairfield, USA; 2 Internal Medicine, University Malaya Medical Centre, Kuala Lumpur, MYS

**Keywords:** coinfection, thrombocytopenia, acute liver injury, dengue fever, covid-19 pneumonia

## Abstract

COVID-19 infection is caused by the severe acute respiratory syndrome coronavirus 2 (SARS-CoV-2), which was declared a pandemic in 2020. Dengue fever (DF) is caused by the dengue virus (DENV) from the *Flaviviridae* family and is transmitted via the bite of the female *Aedes* *aegypti* mosquito. COVID-19 pneumonia and dengue fever coinfection is a relatively difficult diagnosis to be established considering the similarities in the clinical manifestation of both diseases. I hereby report an unusual case of dual diagnosis involving COVID-19 pneumonia and dengue fever (DF) on the same day of presentation to the hospital.

A 62-year-old male presented to the emergency department with a fever of six days duration associated with chills, rigors, arthralgia, myalgia, and a generalized pinpoint rash over the chest and abdomen. He had contact with a worker who recently tested positive for COVID-19. However, his vital signs were stable with peripheral capillary oxygen saturation (SPO2) of 99% under room air. Laboratory investigations showed polycythemia, increased hematocrit levels, and thrombocytopenia. Liver function tests showed evidence of acute hepatitis. Otherwise, the basic metabolic panel and coagulation profile were normal. Viral screens for hepatitis B, hepatitis C, and human immunodeficiency virus (HIV) were negative. The posterior-anterior chest radiograph of the patient showed ground glass opacity in both middle and lower zones of the lungs, which is mostly peripheral with preservation of lung markings.

The diagnosis was confirmed by a positive SARS-CoV-2 polymerase chain reaction (PCR) test with a cycle threshold (CT) value of 19.97 and positive immunoglobulin M (IgM) and immunoglobulin G (IgG) titers on the dengue serology panel on the same day of testing. Predisposing risk factors were chronic medical illnesses (type 2 diabetes mellitus, hypertension, and ischemic heart disease) and exposure to probable COVID-19-infected individuals. The patient fully recovered after treatment with oral paracetamol 1 g four times a day for five days and an intravenous drip of 0.9% sodium chloride for 24 hours.

## Introduction

COVID-19 infection is caused by the novel coronavirus SARS-CoV-2, which was initially reported in Wuhan, China, and declared a pandemic by the World Health Organization in 2020 [1]. COVID-19 is commonly transmitted via respiratory droplets, close contact, and fomites, and as of June 2021, there are around 178 million confirmed cases of COVID-19 worldwide [2]. Dengue fever (DF) is caused by the dengue virus (DENV) from the *Flaviviridae* family and is transmitted via the bite of the female *Aedes aegypti* mosquito with around 70% of the global burden of the disease reported in Southeast Asia and the Western Pacific [3]. There is a paucity of cases reported on the coinfection of COVID-19 and dengue virus in a patient, and there is limited data published regarding the clinical presentation and laboratory findings of such a patient. This case report describes a 62-year-old male who presented with symptoms of dengue fever for six days with positive dengue serology and tested positive for COVID-19 on the same day of presentation to the hospital. He recovered with no complications after the inpatient stay.

## Case presentation

A 62-year-old male presented to the emergency department with a fever of six days duration associated with chills, rigors, arthralgia, myalgia, and a generalized pinpoint rash over the chest and abdomen. He had no cough, sore throat, shortness of breath, abdominal pain, vomiting, diarrhea, bleeding tendencies, loss of sense of smell or taste, or retro-orbital pain. He denied participating in recent jungle trekking or water sport activities, and no recent fogging of his residential area was done. He mentioned that he works in a café and that he might have had contact with one of the workers who recently tested positive for COVID-19. Information regarding mask compliance of the patient was unknown. Otherwise, he denies overseas travel and participation in mass gatherings. His past medical history includes type 2 diabetes mellitus, hypertension, and ischemic heart disease with double-vessel disease and angioplasty done more than five years ago. 

On admission, he was febrile with a temperature of 38.8°C, blood pressure of 145/80 mmHg, pulse rate of 91 beats/minute, and peripheral capillary oxygen saturation (SPO2) of 99% under room air. He had generalized, non-blanching pinpoint petechial rash over the chest and abdomen. Respiratory examination showed bibasal crepitations and no lymphadenopathy but was not in acute respiratory distress. Otherwise, examinations of other systems, including cardiovascular, abdominal, neurological, ear, nose, and throat, were normal. 

His full blood count revealed polycythemia with hemoglobin of 18.3 g/dL and an increased hematocrit level of 0.54 L/L with a concomitant decrease in platelet count of 111 x 10^9^/L. Both total white blood cell count and lymphocyte count were normal at 4 x 10^9^/L and 1.58 x 10^9^/L, respectively. However, the liver function test showed evidence of acute liver injury with an increased total bilirubin level of 19 µmol/L, elevated transaminases with alanine transaminase (ALT) level of 110 U/L and aspartate transaminase (AST) level of 77 U/L, and increased gamma-glutamyltransferase (GGT) level of 138 U/L. His coagulation profile, electrolytes, and renal profile were within normal limits.

**Figure 1:**
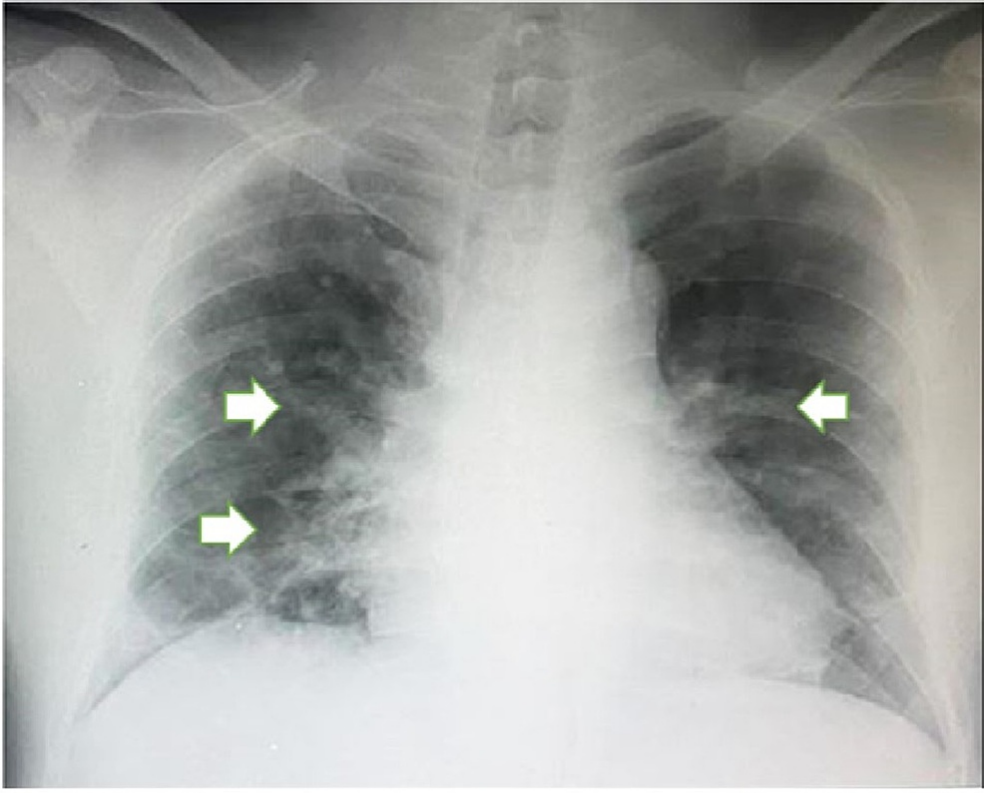
Anterior-posterior chest radiograph of the patient with ground glass opacity in both middle and lower zones (white arrows) with preservation of lung markings.

Viral screens for hepatitis B, hepatitis C, and human immunodeficiency virus (HIV) were negative. A dengue serology panel was sent urgently, and the results showed positive immunoglobulin M (IgM) and immunoglobulin G (IgG) titers with negative NS1 antigen indicating a recent dengue infection with evidence of a past dengue infection due to positive IgG titers. In addition, both nasopharyngeal and oropharyngeal swabs were taken on admission and sent for COVID-19 polymerase chain reaction (PCR) testing that came back positive with a cycle threshold (CT) value of 19.97. COVID-19 PCR testing was done due to his history of possible exposure to a COVID-19-infected individual. His chest radiograph is shown in Figure 1.

He was admitted to the general medical isolation ward and started on an intravenous drip regime of 0.9% sodium chloride of 3 mL/kg/hour for six hours and reduced to 2 mL/kg/hour for the next 24 hours. Intravenous drip was discontinued 24 hours later as he was able to drink plenty of fluids and had a good urine output. He was treated symptomatically for his COVID-19 infection with oral paracetamol 1 g four times a day for five days. He was able to tolerate orally and drank 1-2 L of fluid per day. He also had a good urine output of 2 mL/kg/hour over 24 hours and had no spike in temperature in the ward. His symptoms improved, and he was discharged home after 14 days of quarantine at the hospital. He did not suffer from any complications. Follow-up was done one month later, and he was well and asymptomatic. He was able to do light jogging exercises as well.

## Discussion

COVID-19 pneumonia and DF coinfection in a patient is relatively rare and difficult to diagnose as the clinical symptoms are similar. There are four serotypes of the dengue virus: DEN1, DEN2, DEN3, and DEN4. Patients infected with a single serotype of dengue virus can be infected with another serotype of the dengue virus in the future, resulting in a dengue hemorrhagic fever (DHF), which contributes to increased mortality rates. 

The exact pathophysiological mechanism for coinfection is unknown, but a few case reports have been published regarding this phenomenon. One such hypothesized pathophysiology includes early suboptimal antibody activation leading to continuous inflammation that forms a nidus for persistent replication of the SARS-CoV-2 virus [4]. Another study using animals mentioned that angiotensin II (Ang II) and angiotensin-converting enzyme 2 (ACE2) have a role in the pathogenesis of the coinfection in which the upregulation of Ang II and ACE2 facilitates the entry of SARS-CoV-2 into host cells and the inhibition of both Ang I and ACE reduced the percentage of macrophages expressing DENV2 antigens [5]. 

Regarding the diagnosis of coinfection of both SARS-CoV-2 and dengue virus (DENV), it is extremely difficult for a diagnosis to be made based solely on clinical presentation and requires a more comprehensive workup of the patient through serological tests. This is because both COVID-19 pneumonia and DF present with similar symptoms, such as fever, arthralgia, nausea, and vomiting. At this point, the most common tests used are real-time polymerase chain reaction (RT-PCR) for SARS-CoV-2 and IgM, IgG, and nonstructural protein 1 (NS1) antigen screen for DF. Although ELISA NS1 antigen kits are the most sensitive to diagnose DF, it is usually positive from the onset of fever until day 9 of illness, and IgM levels tend to rise after day 5 of illness [6].

A study by Wang et al. showed that out of 320 patients with DF, serology testing using NS1/IgM gave better performance in detecting acute dengue, with an overall sensitivity of 88.65% and specificity of 98.75% when used. However, the incidences reported of false-positive dengue IgM in patients confirmed to have COVID-19 can be attributed to the possibility of similar antigenic properties between DENV and SARS-CoV-2, which can cause the formation of anti-DENV antibodies when exposed to SARS-CoV-2 [7,8]. I would recommend testing the patient for both COVID-19 and DF if high in suspicion and if the healthcare facility has adequate testing facilities for the tests mentioned above.

The ability to differentiate a coinfection with SARS-CoV-2 and DENV is pertinent to be able to proceed with the correct management. In this case report, the patient was diagnosed with category 3 COVID-19 infection, which shows that the patient is symptomatic with evidence of pneumonia with dengue fever. As per the hospital’s protocol, only confirmed category 4 (hypoxemia with pneumonia) and category 5 (severe acute respiratory distress syndrome) COVID-19 infection will be started on treatment as these patients require supplemental oxygen. Therefore, this patient is only treated for his symptoms.

## Conclusions

In conclusion, the diagnosis of coinfection between SARS-CoV-2 and DENV remains a challenge to physicians, especially in dengue-endemic countries, but prompt detection and treatment result in significantly low rates of mortality and morbidity. If in doubt of a diagnosis involving either COVID-19 pneumonia or DF, it is encouraged to send diagnostic serology (NS1 antigen, dengue IgM, dengue IgG, and COVID-19 RT-PCR) for both conditions to reach a conclusion. The prompt treatment of the coinfection will reduce the risk of hypovolemic shock and acute respiratory distress syndrome in the patient.
